# Quantification of Enzyme–Inhibitor Pharmacological Actions

**DOI:** 10.3390/ijms27219565

**Published:** 2026-10-26

**Authors:** Patrick Masson, Tatiana Pashirova

**Affiliations:** 1 Institute of Fundamental Medicine and Biology, Kazan Federal University, 18 Kremlyovskaya St., 420008 Kazan, Russia; pashirova@iopc.ru; 2 Arbuzov Institute of Organic and Physical Chemistry, FRC Kazan Scientific Center of RAS, Arbuzov Str. 8, 420088 Kazan, Russia

**Keywords:** half maximal inhibitory concentration (*IC*_50_), inhibition constant (*K*_i_), reversible inhibitor, irreversible inhibitor, slow-binding inhibitor, tight-binding inhibitor, allosteric inhibitor

## Abstract

Quantitative measurement of enzyme–drug interactions has become a key step in the process of drug discovery. More and more studies refer to the determination of the operational index, *IC*_50_, the half-maximal inhibitory concentration, from dose–response curves, instead of the determination of the exact dissociation constant, *K*_i_, a true thermodynamic parameter that measures the affinity and provides information on binding mechanisms. Although knowledge of *IC*_50_ is useful for rapid screening and comparison of new molecules, it depends on experimental conditions, in particular substrate and enzyme concentration, type of inhibitor, and incubation time with inhibitor. Thus, *IC*_50_ values can be misleading in cases of allosteric inhibition, partial inhibition, slow-tight binding and progressive inhibition (covalent inhibition). As a consequence, the relationships between *IC*_50_ and *K*_i_ strongly depend on catalytic and inhibition mechanistic models and experimental conditions. The present review provides relationships between *IC*_50_ and *K*_i_ for simple and complex mechanisms of enzyme inhibition and highlights the interest of a thorough analysis of binding processes and irreversible reactions with inhibitors to describe the potentialities of new pharmacologically active compounds, acting either on Michaelian or non-Michaelian enzymes.

## 1. Introduction

Inhibitors of enzymes, with receptor ligands, are certainly the most important physiologically active molecules with respect to their pharmacological and toxicological properties.

At this point we have to consider two classes of compounds: irreversible inhibitors and reversible inhibitors. Their pharmacological activities are dependent on binding affinity and/or reactivity for specific physiological targets. Affinity of molecules is conveniently measured by the *IC*_50_ index (half-maximal inhibitory concentration), as a proxy of real binding affinity, i.e., *K*_i_, the dissociation constant of enzyme–inhibitor complex, that is the thermodynamic parameter directly related the Gibbs free energy of interaction ($∆G_{int}=RTLnK_{i}$), as it was pointed out [1]. Moreover, *IC*_50_ knowledge has numerous limitations that can lead to erroneous judgment, particularly in the case of non-classical enzyme inhibitors (e.g., partial inhibitors, tight-binding inhibitors) and non-Michaelian behavior of enzymes. These limitations may impair evaluation of both efficacy and safety of enzyme inhibitors as new drugs [1]. The present review highlights crucial binding and kinetic issues in early steps of drug discovery and advocates for a return to rigorous analyses of the binding affinity and inhibition kinetics of enzymes.

In general, binding of reversible inhibitors is very fast, as is the binding of substrates, often in the sub-millisecond range [2]. However, in some cases, binding/dissociation kinetics are slow, leading to a long establishment of steady-state and long residence time on targets [3]. With irreversible inhibitors, inhibition is characterized by formation of a covalent bond between enzyme and inhibitor. This process is time-dependent, and its strength depends on the bimolecular rate constant [4].

Thus, the present review deals with experimental approaches implemented and formal analysis of experiments for quantification of binding, kinetics and strength of reversible and irreversible inhibitors.

Although QSAR methods, in particular 4D-QSAR calculations now possible, opened the door to advanced QSAR methodologies (5D-, 6D-, 7D-QSAR) for highly reliable predictions of drug properties, targeting more complex systems [5,6,7], as well as new in silico approaches, including molecular docking/molecular dynamics simulations, virtual screening [8,9,10], QM/MM [10], and the use of machine learning and artificial intelligence [11,12,13] that are very powerful, we will exclusively focus on classical in vitro approaches for investigation of specific inhibitors. These methods can be implemented for HTP screening of molecule libraries. Classical in vitro methods include mostly steady-state kinetic analysis of reversible and irreversible enzyme inhibition, as described in all enzyme textbooks, but also new methods for investigating binding affinity such as isothermal calorimetry and surface plasmon resonance, as they are now implemented for drug discovery [14]. In certain particular cases of enzyme inhibition, X-ray structure analysis and molecular modeling approaches have to work together with enzyme kinetic analysis to describe binding mechanisms accurately [15]. Information provided by in vitro binding and kinetic studies is essential in early steps of drug discovery, as it has been masterfully shown not only for the discovery of simple reversible inhibitors of Michaelian enzymes but also for allosteric modulators, tight-binding inhibitors, covalent inhibitors, and effectors of encapsulated enzyme systems [16]. Thus, we will focus on the formal kinetic analysis and theoretical foundations for processing data from in vitro experiments. Of course, quantitative data obtained by using classical in vitro approaches must be compared with predictive in silico methods and, ultimately, validated by in vivo data on cell cultures and animals.

## 2. Reversible Enzyme Inhibitors

### 2.1. Classical Fast Reversible Inhibition

**Scheme 1:**
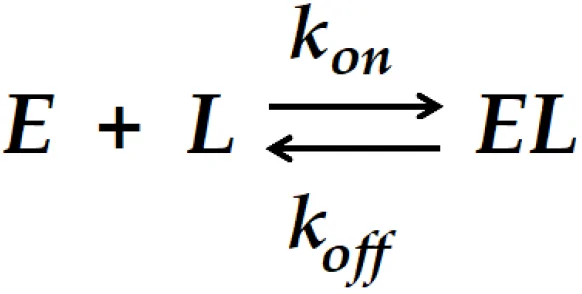
Simplest scheme for reversible binding of a ligand with an enzyme.

The interaction of enzymes (*E*) with ligands (*L*) can be quantified by determining the dissociation constant, *K*_D_, of the enzyme–ligand complex, *EL* (Scheme 1), in which *k*_on_ is the rate constant of ligand binding to *E,* and *k*_off_ is the rate constant of complex EL dissociation.

The dissociation constant *K*_D_ is the ratio *k*_off_/*k*_on_.

(1) $$K_{D}=\frac{k_{off}}{k_{on}}=\frac{\left[E\right]\left[L\right]}{\left[EL\right]},$$

In this context, *L* can be either inhibitor (*I*) or activator (*A*). It can bind to active centers or to modulator and allosteric sites.

**Scheme 2:**
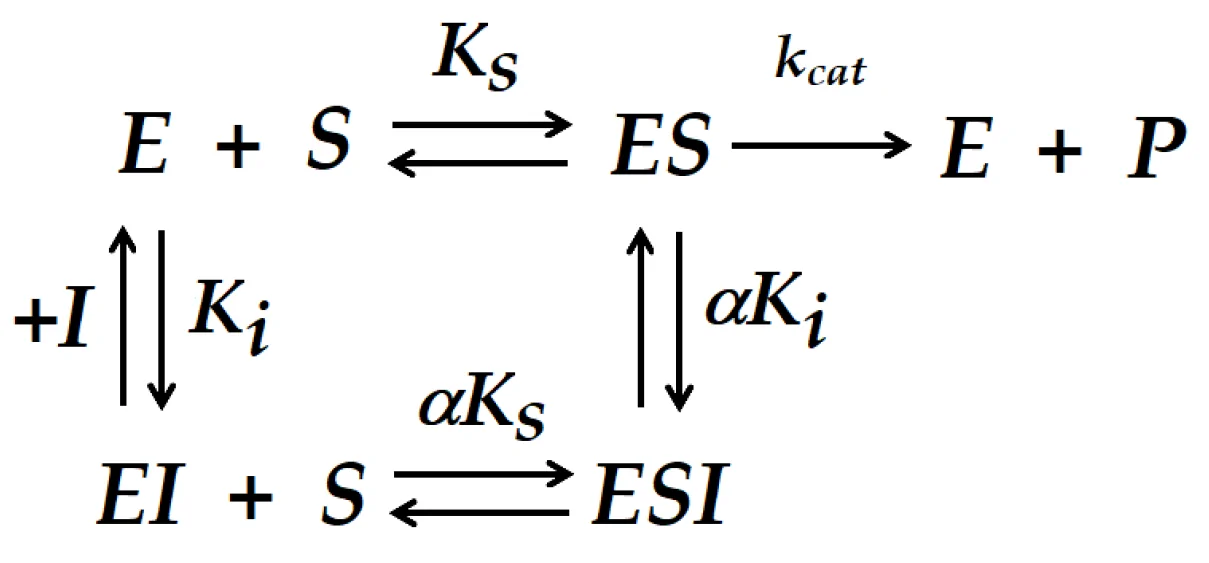
The different cases of reversible inhibition.

Taking *I* as a reversible inhibitor, several cases must be considered. Scheme 2 shows the different cases of reversible inhibition after reaching steady state.

The different possibilities of fast reversible inhibition of the simplest Michaelis–Menten enzyme catalytic mechanism.

Competitive inhibitors bind the free enzyme (*E*) exclusively. Non-competitive inhibitors bind equally to *E* and *ES*. Mixed inhibitors bind to both enzyme forms with different affinities (*K*_i_ ≠ α*K*_i_). Uncompetitive inhibitors bind to the *ES* complex.

In Scheme 2, *K*_s_ is the dissociation constant of the enzyme–substrate complex (ES), and *K*_i_ is the dissociation constant of the enzyme inhibitor complex (EI). The coefficient *α* indicates the change in K_S_ and K_i_ due to binding of *I*. For pure competitive inhibition, *α* = ∞ and *K*_i_ ≪ *αK*_i_; for non-competitive inhibition, *α* = 1 and *K*_i_ = *αK*_i_; for uncompetitive inhibition, *K*_i_ = ∞ and *αK*_i_ ≪ *K*_i_; for competitive mixed-type inhibition, *α* > 1 and *K*_i_ < *αK*_i_; for uncompetitive mixed-type inhibition, *α* < 1 and *αK*_i_ ≪ *K*_i_. Therefore, the classical way to measure the strength of enzyme inhibitors is to measure *K*_i_, i.e., the *K*_D_ of the enzyme–inhibitor complex.

*K*_D_*= K*_i_ = [*E*][*I*]/[*EI*], (2)

**Figure 1:**
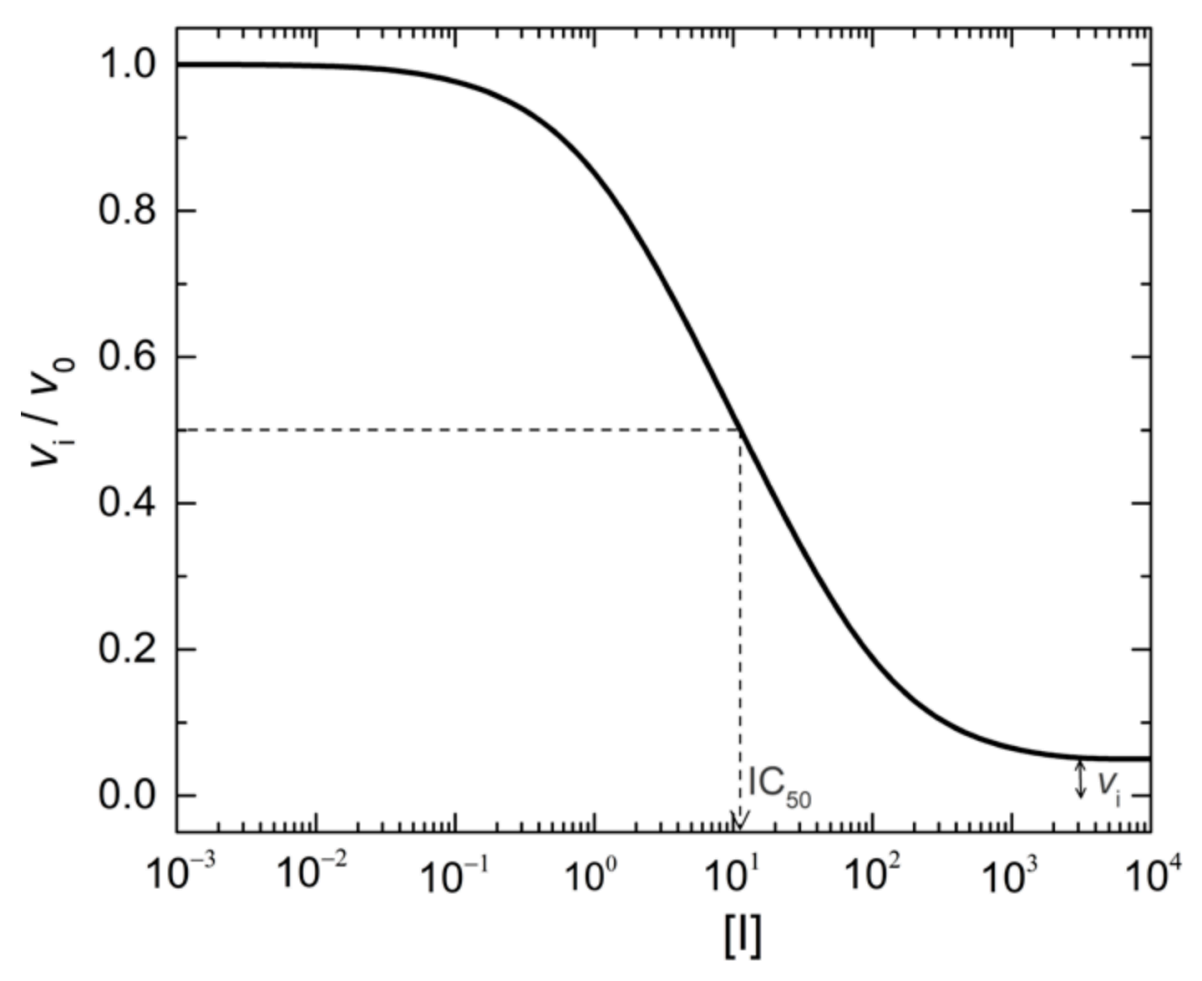
Theoretical dose–response curve, *v*_i_/*v*_0_ = f([*I*]). *v*_i_*/v*_0_ = 0.5 is obtained for [*I*] = *IC*_50_. The residual enzyme activity (*v*_i_) at high [*I*] indicates partial reversible inhibition.

Alternatively, the index *IC*_50_ was proposed; *IC*_50_ corresponds to the inhibitor concentration causing 50% inhibition of the enzyme activity. Thus, dose–response curves for enzyme inhibition can be conveniently drawn graphically by plotting the remaining fractional activity of the enzyme (*y* = *v*_i_/*v*_0_) as a function of inhibitor concentration [*I*] (Figure 1). At [*I*] = 0, *v* = *v*_0_, *y* = 1, and at the highest [*I*], the residual activity (*v*_i_) reaches *y*_i,min_ (Equation (3)). For most reversible inhibitors, at high [*I*], *y*_i,min_ → 0 [17]:

(3) $$\frac{v_{i}}{v_{0}}=y_{i,min}+\frac{1-y_{i,min}}{\left[1+\left(\frac{\left[I\right]}{{IC}_{50}}\right)\right]},$$

Under these conditions, the inhibitor concentration at the transition mid-point, *v*_i_/*v*_0_ = 0.5, corresponds to *IC*_50_. However, if *y*_i,min_ reaches a finite limit > 0, it means that there is partial inhibition. Partial reversible inhibitors (PRI) do not fully inhibit the enzyme even at the highest concentrations; i.e., the complex ESI in Scheme 2 is productive. Then, for partial reversible inhibitors, dose–response curves do not reach zero at high inhibitor concentration (Figure 1). The height of asymptotic activity (*v*_i_) at high [*I*] corresponds to the residual fractional activity [18]. The case of PRI shows a limit in the use of *IC*_50_ for quantification of enzyme–inhibitor relationships.

The relationships between *K*_i_ and *IC*_50_ were first analyzed by Cheng and Prusoff [19], who derived equations describing the relationships between enzyme catalytic parameter ([*S*]/*K*_m_) and inhibition parameters, *K*_i_ and *IC*_50_, for the different types of fast reversible inhibitors, operating under first-order conditions, i.e., [*E*] ≪ [*I*] (Scheme 2, Table 1).

As seen in Table 1, *IC*_50_ depends on substrate affinity (*K*_m_) and substrate concentration, except for non-competitive inhibitors. Thus, *IC*_50_ is largely dependent on the ratio [*S*]*/K*_m_.

A thorough analysis of the different inhibition mechanisms of enzymes following the Michaelis–Menten mechanism led to more complex relationships [20], demonstrating that for all inhibitors *IC*_50_ ≥ *K*_i_ and *IC*_50_ ≥ [*E*]_t_/2. In the particular cases of tight-binding inhibitors [21], partially reversible inhibitors [22], and inhibition of allosteric enzymes, new relations were derived. The following sections deal with these particular cases.

### 2.2. Slow-Binding and Tight Binding Inhibition

For slow-binding inhibitors and tight-binding inhibitors, the respective concentrations of enzyme and inhibitors are important. For slow-binding inhibitors acting under first-order conditions ([*E*] ≪ [*I*]), the steady-state equilibrium between enzyme and inhibitor is established after a lag time (*τ*) that could be of several minutes (Equation (4))

(4) $${\left[P\right]}_{t}=v_{\mathrm{ss}}t+\frac{\left(v_{0}-v_{\mathrm{ss}}\right)\left(1-\exp\left(-k_{\mathrm{obs}}t\right)\right)}{k_{\mathrm{obs}}}$$

In Equation (4), the lag time is the reciprocal of the first-order rate constant, *k*_obs_ = 1/τ; *v*_0_ is the initial rate of the enzyme in the absence of inhibitor, and *v*_ss_, the rate after establishment of steady-state of inhibition [23,24].

Lag time is related to the dissociation rate constant of inhibitor from enzyme and measures the residence time of inhibitor on its target. Long residence times cause temporal discordances between pharmacokinetics and pharmacodynamics [25]. Slow-binding inhibitors that display in vivo long residence times on their targets inhibit these enzymes at doses far below *IC*_50_. For these inhibitors, apparent *IC*_50_ can lead to large underestimation of the inhibitory potency. It was the case for the anti-Alzheimer slow-binding inhibitor galantamine [26]. Thus, for slow-binding inhibitors, *IC*_50_ must be determined at equilibrium after the steady-state in the presence of inhibitor (*v*_ssi_) has been reached.

Tight-binding inhibitors have a strong affinity for enzymes; they react under quasi-stoichiometric conditions. For tight-binding inhibitors, Equation (4) becomes Equation (5) [27]:

(5) $$\left[P\right]=v_{\mathrm{ss}}t+\frac{\left(v_{0}-v_{\mathrm{ss}}\right)\left(1-\gamma \right)}{k_{\mathrm{obs}}\gamma }\ln\left\{\frac{\left[1-\gamma exp(-k_{\mathrm{obs}}t\right]}{1-\gamma }\right\}$$

in which γ is a function of *v*_ss/_*v*_0_,

(6) $$\gamma =\frac{\left[E\right]}{\left[I\right]}{\left(1-\frac{v_{ss}}{v_{0}}\right)}^{2}$$

Thus, in practice, *v*_0_, *v*_ss_ and *k*_obs_ are determined from Equation (5). It is important to note that γ is a function of *v*_0_, *v*_ss_ and [*I*], and thus, must not be considered as an independent parameter. However, for tight-binding inhibitors, the first-order condition requirement used in Cheng–Prusoff equations is not respected when [*E*] and [*I*] are of the same order (Equation (6)), and *IC*_50_ depends on both *K*_i_ and total enzyme concentration. Therefore, *IC*_50_ values are misleading if there is tight tight-binding inhibition. With tight-binding inhibitors, it is important to take into account the free inhibitor depletion due to binding to the enzyme. Starting from the generalized Henderson’s equation [28], in which ${\left[E\right]}_{t}$ stands for the total enzyme concentration, a new relationship can be established Equation (7):

(7) $$\frac{{\left[I\right]}_{t}}{\left(1-\frac{v_{i}}{v_{0}}\right)}={\left[E\right]}_{t}+\left(\frac{\left[S\right]+K_{m}}{\frac{K_{m}}{K_{i}}+\frac{\left[S\right]}{{\alpha K}_{i}}}\right)\frac{v_{0}}{v_{i}}$$

Cha-derived equations give *IC*_50_ for the different types of tight-binding inhibitors [21]. The apparent inhibition constants, *K*_i,app_, at equilibrium, for the different types of tight-binding inhibition, as in Scheme 2, are composed of two terms: [*E*]_t_/2 *plus* the *K*_i_ term function of [*S*] and *K*_m_, depending on the type of inhibition (Table 1). Then, the relationships between *K*_i_ and *IC*_50_ for tight binding inhibitors imply that values of *IC*_50_ in Table 1 are simply replaced by *IC*_50_ − [*E*]_t_/2, i.e., *IC*_50_ = [*E*]_t_/2 + $K_{i,app\;}.$

Alternatively, the Morrison’s quadratic equation for enzyme kinetics at high enzyme concentration [29,30] can also be used for determination of $K_{i,app\;}$ and [*E*]_t_ [31] as a function of ([*I*]), in which [*I*] is the total inhibitor concentration and assuming rapid-equilibrium binding (Equation (8)):

(8) $$\frac{v_{i}}{v_{0}}=\;\frac{{(\left[E\right]}_{t}-\left[I\right]-K_{i,app})+\sqrt{{\left({\left[E\right]}_{t}-\left[I\right]-K_{i,app}\right)}^{2}+4{\left[E\right]}_{t}K_{i,app}}}{2{\left[E\right]}_{t}}$$

### 2.3. Reversible Inhibition of Allosteric Enzymes

In the case of allosteric inhibitors, the slope of the dose–response curves is affected and indicates cooperativity in the transition. The fractional enzyme activity as a function of [*I*] is Equation (9):

(9) $$\frac{v_{i}}{v_{0}}=\frac{1}{1+{\left(\frac{\left[I\right]}{{IC}_{50}}\right)}^{n_{H}}}$$

Equation (9) can be expressed as the percentage of inhibition Equation (10),

(10) $$Inhib\%=\frac{100}{1+{\left(\frac{{IC}_{50}}{\left[I\right]}\right)}^{n_{H}}}$$

**Figure 2:**
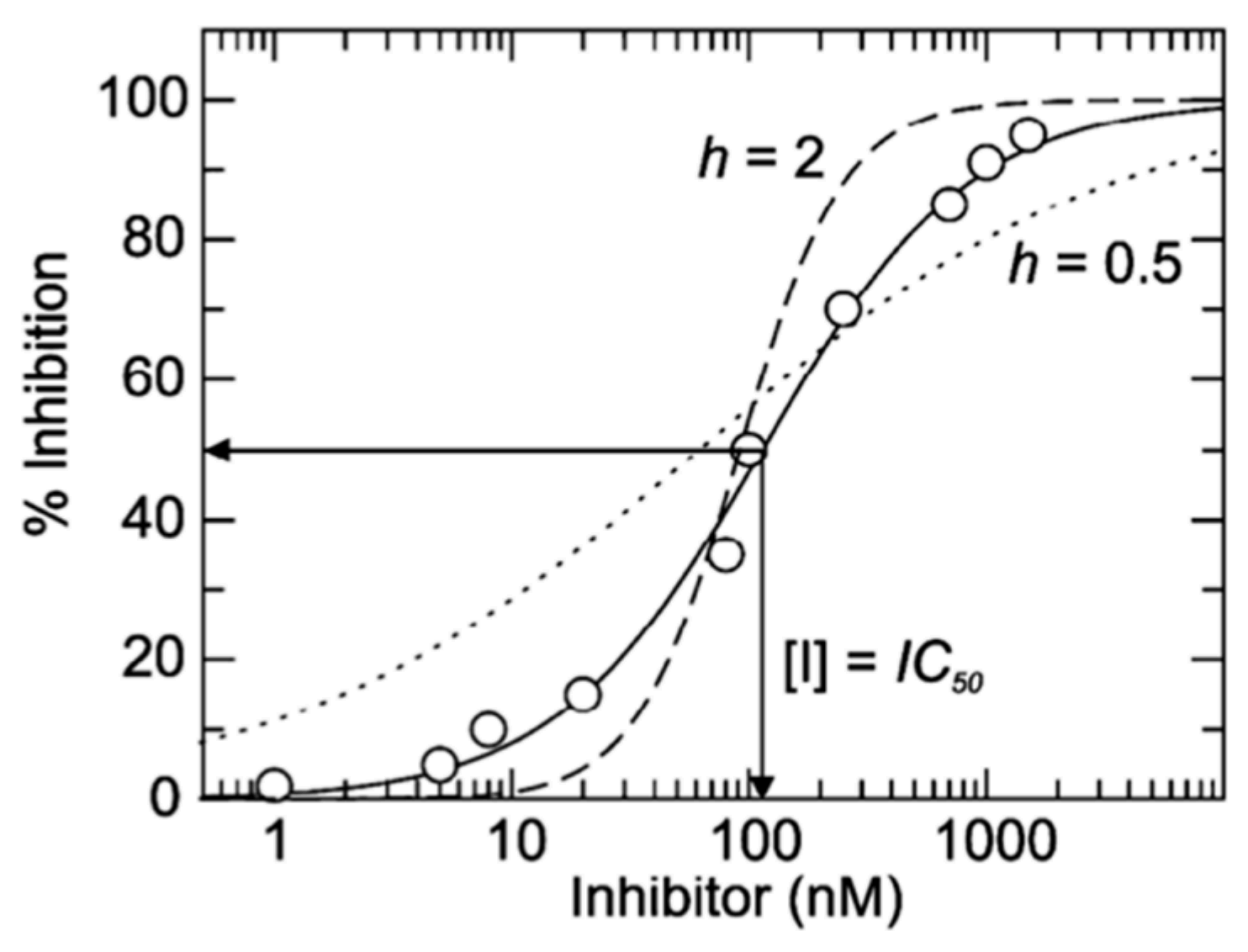
Dose–response curves for a Michaelian enzyme (*n* = h = 1, continuous curve) and allosteric enzymes, *n* = h > 1 and *n* = h < 1 (reproduced from PJ Tonge [33].

When there is no cooperativity, the Hill coefficient (n_H_) in Equation (9) is equal to 1, and Equation (9) is similar to Equation (3). n_H_ > 1 indicates positive cooperativity and n_H_ < 1, negative cooperativity (Figure 2) [32].

However, the equations of Cheng and Prusoff (Table 1) are not operative because the cooperativity and conformational transitions impair the linear relationships between [*S*] and *IC*_50_. Also, it is important to remember that the Hill coefficient is a phenomenological parameter that provides no information about the allosteric mechanism. A phenomenological analysis is, however, possible regardless of knowledge of the allosteric mechanism. Thus, for an allosteric enzyme governed by the Hill coefficient, *n*_H_, the velocity in the absence of inhibitor can be described by a modified form of the Michaelis–Menten rate equation (Equation (11)):

(11) $$v_{0}=\;\frac{V_{max}{\left[S\right]}^{n_{H}}}{K_{m}^{n_{H}}+{\left[S\right]}^{n_{H}}}$$

(for allosteric enzymes, *K*_m_ is sometimes written *K*_0.5_).

Given that for [*I*] = *IC*_50_, *v_i_* = *v*_0_/2, the Cheng and Prusoff equations can be modified accordingly. Then, for competitive allosteric inhibitors, the higher the [*S*], the lower the inhibitor efficacy (Equation (12)):

(12) $$K_{i}=\;\frac{{IC}_{50}}{1+\;{\left(\frac{\left[S\right]}{K_{m}}\right)}^{n_{H}}}$$

For uncompetitive allosteric inhibitors, the situation is opposite: the higher the [*S*], the higher the inhibitor efficacy (Equation (13)):

(13) $${\alpha K}_{i}=\;\frac{{IC}_{50}}{1+\;{\left(\frac{K_{m}}{\left[S\right]}\right)}^{n_{H}}}$$

For pure non-competitive inhibitors, [*S*] does not affect the binding affinity and *K*_i_ does not depend on the Hill coefficient:

(14) $$K_{i}=\;{IC}_{50\;}$$

For allosteric mixed-type inhibitor, inhibitor binds on *E* and *ES*, depending on α (Equation (15)). If *α* >1, *I* binds mainly to *E.* If *α* <1, *I* binds mainly to *ES*.

(15) $$K_{i}=\frac{{IC}_{50}}{\left(1+{\left(\frac{\left[S\right]}{K_{m}}\right)}^{n_{H}}\right)/\left(1+\frac{1}{\alpha }{\left(\frac{\left[S\right]}{K_{m}}\right)}^{n_{H}}\right)}$$

The phenomenological analysis does not provide information how inhibitors interact with enzymes. However, Chritopoulos and Kenakin [34] pointed out that in the case of allosteric inhibitors that bind at a site different from the active site (substrate binding site), the Cheng and Prusoff relations are modified; these inhibitors, forming a ternary complex *ESI*, affect the substrate affinity by a cooperativity factor β, higher than 1 for positive cooperativity or less than 1 for negative cooperativity (Equation (16)):

(16) $${IC}_{50}=\;K_{i}\frac{\left(1+\frac{\left[S\right]}{K_{m}}\right)}{\left(1+\;\frac{\beta \left[S\right]}{K_{m}}\right)}$$

Then, if the allosteric mechanism is known, a thorough analysis can be performed. Each solution depends on the considered mechanism, e.g., MWC (Monod–Wyman-Changeux), KNF (Koshland–Nemethy–Filmer). In the case of the MWC model, considering the transition between *R* (relaxed) and *T* (tensed) states for an oligomeric enzyme composed of *n* identical subunits, *IC*_50_ depends on the allosteric constant *L*_0_ = [*T*]_0_/[*R*]_0_ and [*S*]. If the inhibitor binds exclusively to the *T* form (*K*_i_ = *K_i_*_,T_), then an expression of *IC*_50_ can be derived (Equation (17)):

(17) $${IC}_{50}=\;K_{i}\left[{\left(\frac{\delta \;}{\gamma L_{0}}+2\right)}^{1/n}-1\right]$$

in which δ and γ are related to substrate binding on *R* and T forms, respectively,

(18) $$\delta ={\left(1+\left[S\right]/K_{R}\right)}^{n}$$

(19) $$\gamma ={\left(1+\left[S\right]/K_{T}\right)}^{n}\;$$

If the substrate does not bind to the *T* state ($K_{T}\to \infty$) while the allosteric inhibitor exclusively binds to the *T* state, in the presence of *I*, the R⇌T equilibrium is shifted towards *T* [35], and the enzymatic rate in the presence of allosteric inhibitor is Equation (20):

(20) $$v_{i\;}=\frac{V_{max}\frac{\left[S\right]}{K_{R}}{\left(1+\frac{\left[S\right]}{K_{R}}\right)}^{n-1}}{{\left(1+\frac{\left[S\right]}{K_{R}}\right)}^{n}+\;{\left[L\right]}_{0}{\left(1+\frac{\left[I\right]}{K_{i}}\right)}^{n}}$$

Because at *IC*_50_ the velocity is equal to *v*_0_/2, it follows that

(21) $${IC}_{50}=K_{i}\left[{\left(\frac{{\left(1+\frac{\left[S\right]}{K_{R}}\right)}^{n}}{{\left[L\right]}_{0}}+2\right)}^{1/n}-1\right]$$

Thus, when the majority of *T* state forms are inhibited, Equation (21) is reduced to

(22) $${IC}_{50}\approx K_{i}\;(2^{\frac{1}{n}}-1)$$

This equation holds when most of the enzyme is in the T state (*L*_0_ ≫ δ. Equation (22) is similar to Equations (16) and (17) derived by Lazareno and Birdsall [36] for antagonist binding on receptors. For a tetrameric enzyme (*n* = 4), *IC*_50_ is close to 0.2*K*_i_. Therefore, due to cooperativity between subunits, the inhibition is much stronger for an allosteric enzyme that obeys the MWC model than for a Michaelian enzyme for which, as written above, *IC*_50_ ≥ *K*_i_.

### 2.4. Practical Considerations for the Study of Reversible Inhibitors

Practically, experiments must be performed under steady-state and first-order conditions, i.e., at very low enzyme concentration, [*E*] ≪ [*I*]. The steady-state conditions are important to check, particularly in the case of slow- and tight-binding inhibitors where the equilibrium can be reached after a long time. For determination of either *K*_i_ or *IC*_50_, the reporter substrate concentrations must be much less than the saturating substrate concentration.

Determination of inhibition constants is generally performed by steady-state kinetic measurements. This implies working at multiple inhibitor concentrations in the presence of several substrate concentrations. The most convenient method was described by Cornish-Bowden [37], using the graphical representations of Dixon (1/*v* vs. [*I*]) at a minimum of three different substrate concentrations, and the Cornish-Bowden plot ([*S*]/*v* vs. [*I*]) under the same experimental conditions. For example, in the case of simple Michaelis–Menten kinetics, [*S*]_1_ < *K*_m_; [*S*]_2_ > *K*_m_; [*S*]_3_ ≫ *K*_m_, a concentration close to the saturating concentration leading to *V*_max_. Cornish-Bowden recommends using [*S*] ranging from 0.2*K*_m_ to 10*K*_m_ [38]. The application of Dixon plot analysis to determination of *IC*_50_ was reviewed by [39].

Because the value of *IC*_50_ depends on the ratio [*S*]/*K*_m_, ideally *IC*_50_ must be determined at different [*S*]. A single [*S*] = *K*_m_ allows HTP screening of inhibitors [40]. For more thorough investigations, regardless of the type of inhibition, Bearne demonstrated what the optimum substrate concentrations are to maximize the differences *v*_0_-*v*_i_ [41]. In the case of competitive inhibitors,

(23) $${\left[S\right]}_{opt}=K_{m}\sqrt{1+\frac{\left[I\right]}{K_{i}}}$$

**Figure 3:**
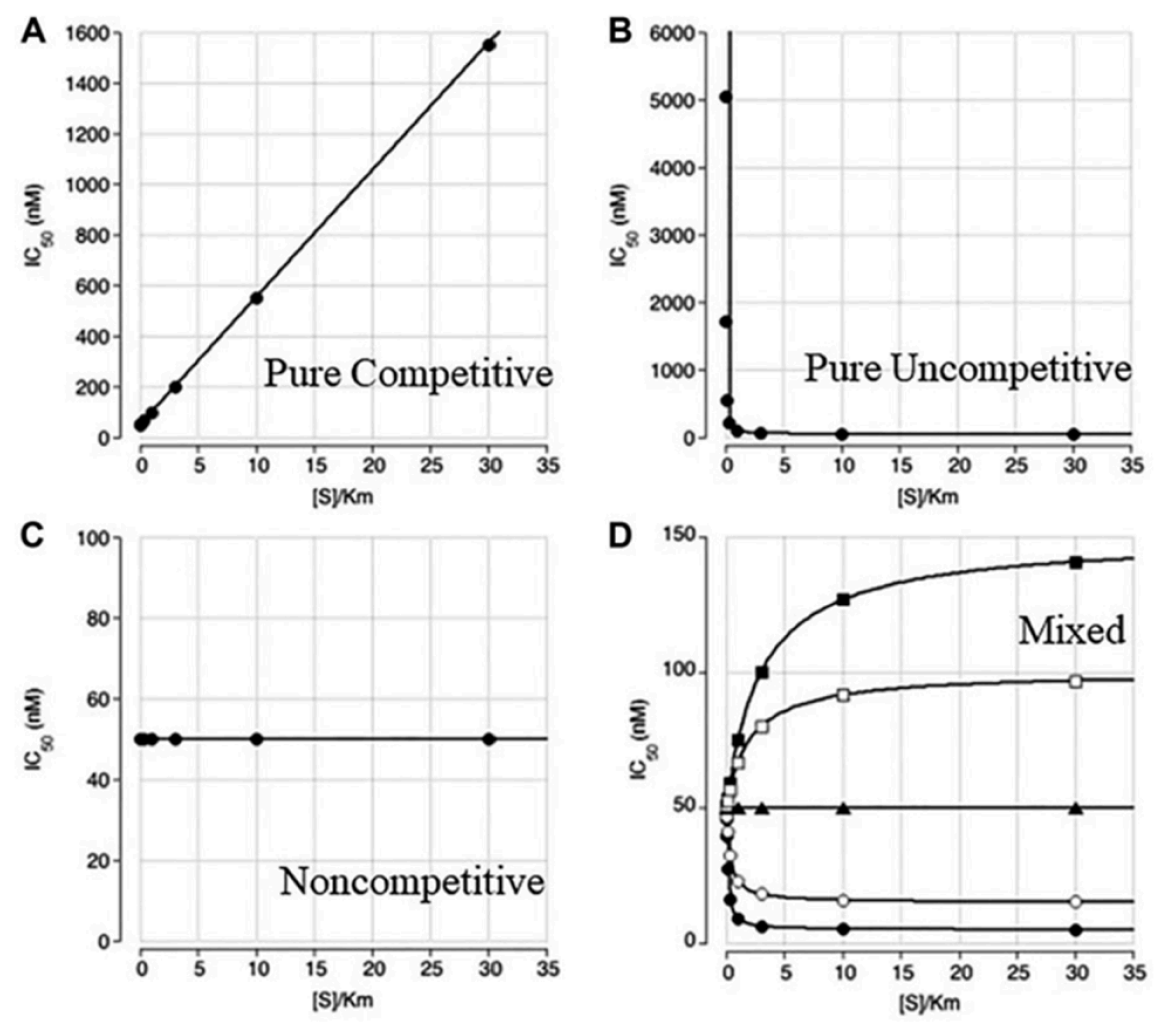
Replot of *IC*_50_ as a function of [S]/K_m_. Reproduced from [42] with permission. (**A**) Pure competitive inhibition. (**B**) Pure uncompetitive inhibition. (**C**) Noncompetitive inhibition. (**D**) Mixed inhibition with α = 0.1 (closed circles), 0.3 (open circles), 1.0 (closed triangles), 2.0 (open squares), and 3.0 (closed squares).

In the case of non-competitive and uncompetitive inhibition, *v*_0_-*v*_i_ increases with [*S*] up to saturating [*S*]; thus, ${\left[S\right]}_{opt}$ around 2 or 3 *K*_m_ is a secure compromise. However, an accurate determination of *IC*_50_ implies working at different values of ratio [*S*]/*K*_m_ [42]. Then, a replot of *IC*_50_ vs. [*S*]/*K*_m_ provides an unambiguous graphical identification of the type of inhibition (Figure 3).

This approach has been recommended for HTP screening of new enzyme inhibitors [43]. For HTP screening of tight-binding inhibitors, Kuzmic et al. [44] developed an automatic method based on the use of Morrison’s equation (Equation (8)); this screening method was further optimized [45]. This approach can be extended to analysis of mixtures of inhibitors, particularly when the inhibitor of interest is contaminated by more potent impurities; Kuzmic et al. proposed a method [46].

## 3. Progressive or Covalent Inhibitors

### 3.1. Kinetics of Irreversible Inhibition

**Scheme 3:**
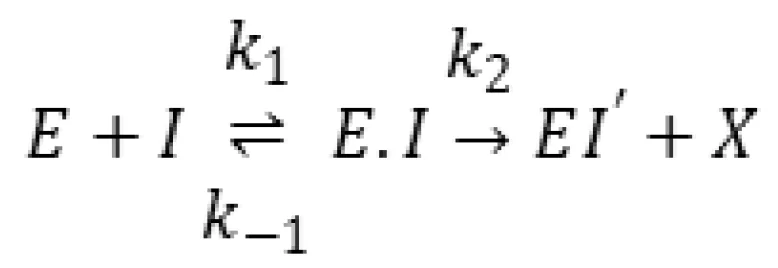
Simplest mechanism for irreversible inhibition of enzymes.

Covalent inhibitors are becoming very important in pharmacology [47,48]. Covalent inhibitors are irreversible enzyme inhibitors. Unlike reversible inhibitors, irreversible inhibition is time-dependent or progressive (Scheme 3). Therefore, *IC*_50_ is time-dependent.

The formation of reversible complex (*E.I*) is followed by an irreversible alkylation step (*EI’*). This chemical step is accompanied by the release of a leaving group (X).

In Scheme 3, *k*_1_ = *k*_on_ is the rate of association of *E* and *I*, *k*_−1_ = *k*_off_ is the rate of dissociation of E.I with (*k*_−1_ + *k*_2_)/*k*_1_ = *K*_i_, and *k*_2_ is the rate of reaction of *E* with *I*. Assuming that equilibrium conditions hold, *k*_2_ ≪ *k*_−1_, then *k*_−1_/*k*_1_ = *K*_i_, and the observed rate of irreversible inhibition, *k*_obs_, is

(24) $$k_{obs}=\frac{k_{1}k_{2}\left[I\right]}{k_{1}\left[I\right]+k_{-1}}=\frac{k_{2}}{1+\frac{K_{i}}{\left[I\right]}}$$

The constants *k*_2_ and *K*_i_ can be determined by non-linear fitting of Equation (24). The ratio *k*_2_/*K*_i_ is the bimolecular rate constant *k*_i_ (M^−1^.min^−1^). If [*I*] ≪ *K*_i_, Equation (24) reduces to Equation (25):

(25) $$k_{obs}=\frac{k_{2}}{K_{i}}\left[I\right]$$

and the exponential decay of product release as

(26) $${\left[P\right]}_{t}=\frac{v_{0}}{k_{obs}}(1-exp(-k_{obs}t))$$

With certain irreversible inhibitors, the covalent step can be preceded by a slow establishment of the complex *E.S* [24]. This contributes to increasing the time-dependency of *IC*_50_. Theoretical analysis and methodology for investigating such inhibitors were recently reviewed [4,49].

**Figure 4:**
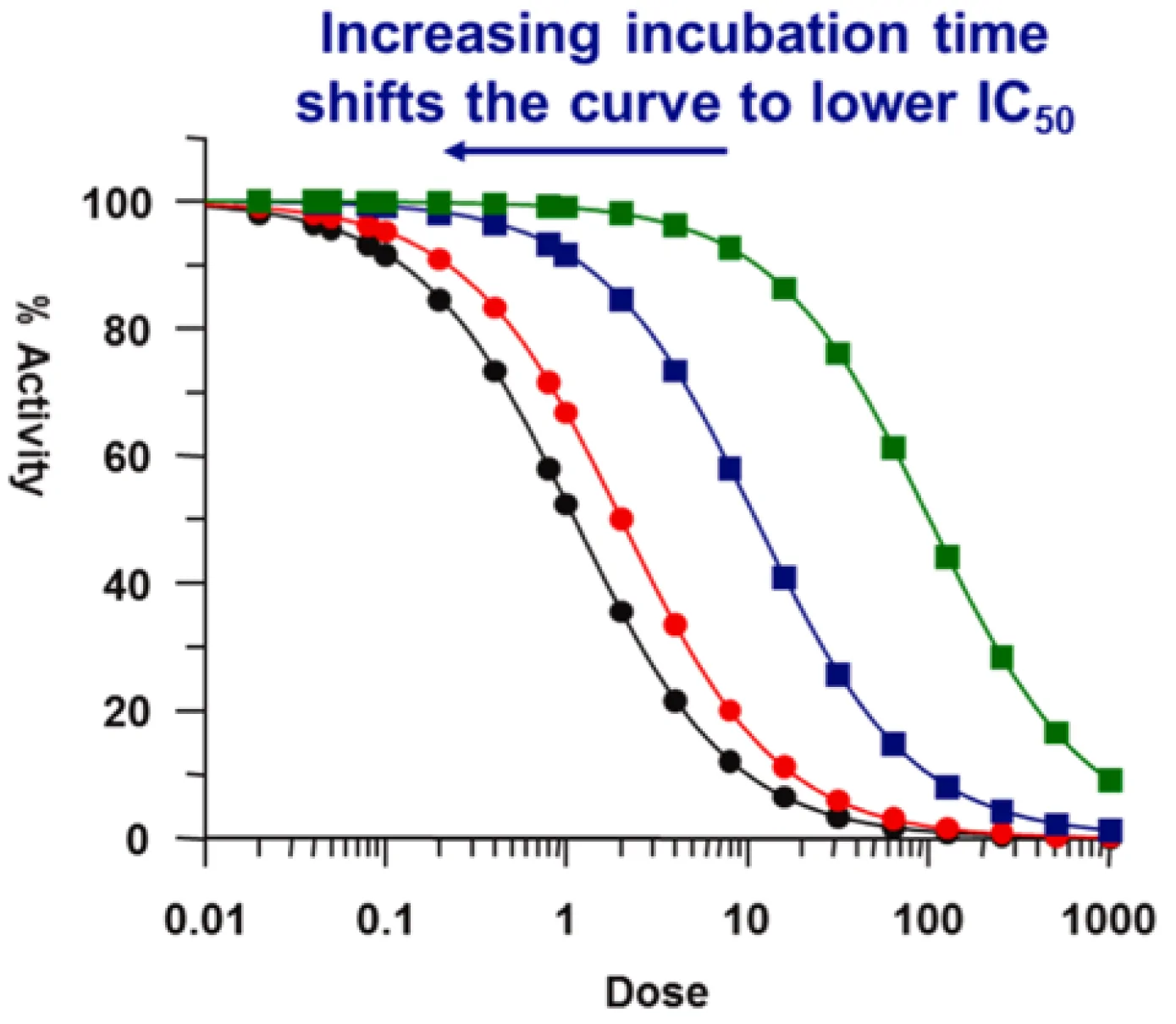
Progressive shift of *IC*_50_ toward ${IC}_{50}^{min}$ as a function of time for covalent inhibitors. Reproduced from [50] with permission.

With irreversible inhibitors, the progressive inhibition does not reach a steady-state after a certain reaction time but continues until all enzyme molecules are inactivated or progressively reaches a plateau if the inhibitor is spontaneously degraded in a concurrent process, e.g., inhibitor hydrolysis, irreversible photo-inactivation, secondary dealkylation of EI’ → EI’’. There is also the possibility that a competing process of spontaneous regeneration of the enzyme EI’ → E takes place. Therefore, when determining *IC*_50_ at fixed time points, *IC*_50,t_, there is a time-dependent change in *IC*_50_ toward the lowest *IC*_50_ (Figure 4). This can be empirically described by Equation (27):

(27) $${IC}_{50(t)}={IC}_{50}^{min}+({IC}_{50}^{t=0}-{IC}_{50}^{min})e^{-kt}$$

In Equation (27), *k* is a phenomenological first-order rate constant, indicating how fast the observed *IC*_50_ values are decreasing with increasing the incubation time in the presence of [*I*]. After a long incubation time, the curve, described by Equation (27), reaches the minimal value of ${IC}_{50}^{min}=$ [*E*]/2 [50] and may, then, go back, in the opposite direction, if spontaneous reactivation occurs.

### 3.2. Practical Considerations for the Study of Progressive Inhibitors

To analyze inhibition of enzymes (*E*) by an irreversible inhibitor (*I*), monitoring of the progressive loss of enzyme activity under first-order conditions, i.e., [*I*] ≫ [*E*] (Scheme 3) must be performed. Exponential or logarithmic plots of activity decay as a function of time for different concentrations of inhibitor allow determination of *k*_obs_ (Equation (28)), and then *k*_i_ and *K*_i_ according to Equations (24) and (25).

(28) $${\left[E\right]}_{t}={\left[E\right]}_{0}e^{-k_{obs}t}$$

in which [E_0_] is the enzyme concentration (or activity) at *t* = 0.

Non-linearity of first-order logarithmic decays in activity can provide important information about molecular or genetic heterogeneity of the enzyme [51].

Progressive spontaneous recovery of inhibited enzyme activity is a first-order process (*k*_s_) that may happen (Equation (29)):

(29) $$\left[E\right]={\left[E\right]}_{0}(1-e^{-k_{s}t})$$

However, this process can be in competition with a first-order secondary post-inhibition irreversible inactivation (*k*_ir_), causing incomplete reactivation of the enzyme [52]. In that case, spontaneous reactivation reaches a plateau ($\left[E\right])$ that allows calculation of the concentration of irreversibly inactivated enzyme ($\left[EIR\right]$ = [*E*_0_] − [*E*]). Then, the height of the plateau, i.e., $\left[E\right],$ allows for determining *k*_ir_, (Equation (30)):

(30) $$\left[E\right]=\frac{k_{s}\left[E_{0}\right]}{\left(k_{s}+k_{ir}\right)}(1-e^{-\left(k_{s}+k_{ir}\right)t}$$

with the concentration of $\left[EIR\right]$ as (Equation (31)),

(31) $$\left[EIR\right]=\frac{k_{ir}\left[E_{0}\right]}{\left(k_{s}+k_{ir}\right)}(1-e^{-\left(k_{s}+k_{ir}\right)t}$$

It follows that that at any time,

(32) $$\frac{\left[EIR\right]}{\left[E\right]}=\frac{k_{ir}}{k_{s}}$$

Then, from [E] and [EIR], the rate constants, *k*_s_ and *k*_ir_, and respective half-times of these competing reactions (*t*_1/2_ = Ln2/*k*) can be calculated. In parallel, apparent *IC*_50_ values must be determined for each incubation time-point until it reaches ${IC}_{50}^{min}.$ If there is spontaneous reactivation or secondary irreversible post-inhibitory reaction, the incubation time must be prolonged, and recovery of activity monitored.

## 4. Conclusions and Future Perspectives

Determination of *IC*_50_ is easy and convenient for rapid screening of enzyme inhibitors. However, it provides no information about the mechanism of inhibition. Determination of inhibition constants (*K*_i_), i.e., the enzyme–inhibitor dissociation constant, from kinetic analysis of the inhibition process provides information on the mechanism of binding and dissociation of inhibitors. In particular, it allows for detecting and analyzing slow binding, tight binding and irreversible processes, as well as partial inhibition mechanisms. We pointed out drawbacks of standard *IC*_50_ measurements in the case of inhibitors that display time-dependent behavior or act as allosteric modulators. Refined inhibition kinetic analysis always allows for discriminating between all classes of inhibitors.

Kinetic analysis of enzyme inhibitor binding/reaction must be completed by structural analyses to establish where (on which site) and how (type of interaction) the inhibitor binds or reacts and affects the catalytic machinery of enzymes. This starts by establishing how many binding/active sites the enzyme processes and whether the enzyme displays molecular and/or genetic polymorphism [51]. This step implies kinetic studies and structural studies (electrophoresis, mass spectrometry, X-ray structure analysis, electron microscopy) and genetic studies to identify isoenzymes and allozymes. After this step, molecular modeling (docking and molecular dynamics for reversible inhibitors plus quantum mechanics/molecular mechanics for covalent inhibitors) will provide information on structure–activity–dynamics relationships. Then, new quantitative structure–activity relationship (QSAR) approaches are future prospects that will allow integration of in vitro and in silico data to improve the predictions of drug binding affinity.

In conclusion, while determination of *IC*_50_ provides very useful information on inhibitors as potential new drugs, determination of *K*_i_ provides important mechanistic information about the mechanism of binding/reaction of the inhibitor. Therefore, in vitro methods must be considered as a foundation. Then, correlations between *K*_i_ and *IC*_50_ and their integration with X-ray structure, molecular modeling, QSAR data and genetic studies provide a more comprehensive “structure–activity–dynamics” framework, allowing for accurate description of the mechanism of action of enzyme inhibitors and rational improvement of the computer design and synthesis of new drugs.

## Figures and Tables

**Table 1:** Relations between *K*_i_ and *IC*_50_ for fast reversible inhibitors.

Type of Inhibitor	*IC*_50_ =	
Competitive	$K_{i}\left(1+\frac{\left[S\right]}{K_{M}}\right)$	if [*S*] ≪ *K_M_*, *K_i_* = *IC*_50_
Non competitive	*K_i_*	*α* = 1
Mixed type	$\frac{\left[S\right]+K_{M}}{\left(\frac{K_{M}}{K_{i}}+\frac{\left[S\right]}{\alpha K_{i}}\right)}$	if *α* = 1, *K*_i_ = *IC*_50_
Uncompetitive	$\alpha K_{i}\left(1+\frac{K_{M}}{\left[S\right]}\right)$	if [*S*] ≫ *K_M_*, *αK_i_* ≈ *IC*_50_

## Data Availability

No new data were created or analyzed in this study. Data sharing is not applicable to this article.

## Abbreviations

The following abbreviations are used in this manuscript:

*IC*_50_	Half-maximal inhibitory concentration
HTP	High throughput
*K_D_*	Dissociation constant
*K*_i_	Inhibition constant
*QSAR*	Quantitative structure–activity relationships
